# Shades of Rage: Applying the Process Model of Emotion Regulation to Managing Anger After Brain Injury

**DOI:** 10.3389/fpsyg.2022.834314

**Published:** 2022-03-18

**Authors:** Jade Abigail Witten, Rudi Coetzer, Oliver H. Turnbull

**Affiliations:** ^1^School of Human and Behavioral Sciences, Bangor University, Bangor, United Kingdom; ^2^The Disabilities Trust, Wakefield, United Kingdom

**Keywords:** acquired brain injury (ABI), anger, emotion regulation (ER), process model of emotion regulation, strategies

## Abstract

Uncontrollable anger is common following an acquired brain injury (ABI), with impaired emotion regulation (ER) being one of the main contributors. Existing psychological interventions appear moderately effective, though studies typically include limitations such as small sample sizes, issues of long-term efficacy, and standardization of content. While ER has been a popular research field, the study of ER for anger management after ABI is less well investigated, and contains few interventions based on the widely used Process Model of ER. This review surveys the efficacy of ER strategies in individuals with ABI, and proposes a novel research design for future interventions. Recommendations are made about: strategy number and type, shared decision-making, approaches to data analysis, and mode of delivery.

## Introduction

It is estimated that 69 million individuals suffer from a Traumatic Brain Injury (TBI) globally each year (Dewan et al., 2018), making it one of the leading causes of death and disability worldwide^1^ (Hyder et al., 2007). In addition to the well-known cognitive impairment, survivors of TBIs and other types of acquired brain injuries (ABI), such as cerebrovascular accidents, experience substantial difficulties with social functioning and employment^2^. These difficulties are further compounded by the presence of aggressive outbursts post-injury (Sabaz et al., 2014).

Uncontrollable anger is common following an ABI (Khan et al., 2003; Caplan et al., 2015; Neumann et al., 2017), with an estimated prevalence of up to 41% during the first 5 years post-injury (Tateno et al., 2003; Baguley et al., 2006; Rao et al., 2009; Roy et al., 2017). Family and loved ones are typically the recipients of uncontrollable expressions of anger, reporting sudden and unpredictable outbursts (Saban et al., 2015). Amongst environmental factors, such responses are likely due to impaired emotion regulation (ER; Arciniegas and Wortzel, 2014; Salas et al., 2014; Caplan et al., 2015; Aboulafia-Brakha et al., 2016; Winter et al., 2018): the ability to modify and control personal experiences and expressions of emotion (Gross, 1998a,^2002^).

The majority of anger management interventions for individuals with ABI (see Byrne and Coetzer, 2016) or mental health disorders (see Ross et al., 2013), focus on *physical* manifestations of aggression. This focus excludes an individual’s *subjective* experience of anger (i.e., emotional outbursts), with important implications for gender differences. An international survey demonstrated *equivalent* levels of anger for men and women, recognizing that women are less likely to transform subjective anger into acts of physical aggression (Özkarar-Gradwohl and Turnbull, 2021). This may explain why domestic abuse is a gendered crime, with implications for treatment eligibility.

### Psychological Interventions for Aggression

Psychological interventions appear moderately effective (*d* = −0.46) in populations with ABI (see Byrne and Coetzer, 2016; Iruthayarajah et al., 2018) and mental health disorders (see Ross et al., 2013; Lee and DiGiuseppe, 2018). The majority of these interventions are based on cognitive behavioral therapy (CBT), which include several limitations.

*Small sample size* (e.g., single case studies; *n* = 1) is a common limitation (Alderman et al., 2013; Ross et al., 2013; Byrne and Coetzer, 2016; Iruthayarajah et al., 2018). Studies in populations with mental health disorders contain larger samples than those with ABI. Ross et al. (2013) reported samples ranging from 3 to 290, while Byrne and Coetzer (2016) and Iruthayarajah et al. (2018) reported ABI samples ranging from 1 to 52. While an adequate sample size is necessary for scientific rigor, the inherent nature of recruiting from clinical populations (and especially those with ABI), makes this a challenging limitation to overcome (e.g., see Armstrong et al., 2020).

The *long-term efficacy* of interventions is also under-investigated, partly because not all studies include follow-up assessments (Byrne and Coetzer, 2016; Iruthayarajah et al., 2018). The majority of studies that *do* report follow-up data do *not* report therapeutic efficacy over time (Ross et al., 2013; Byrne and Coetzer, 2016), meaning that potential gains are not measured.

The *standardization of interventions* is another issue (e.g., Ross et al., 2013; Byrne and Coetzer, 2016). Ross et al. (2013) report differences in the CBT content across studies (i.e., standard CBT versus additional study-specific components), as well as differences in dosage or intensity (i.e., hours versus days), and modality (i.e., individual versus group), of treatments. Some interventions encourage participant involvement in the rehabilitation process (e.g., see McClain, 2005). Mode of administration varies across settings, with an increase in virtually administered services since the COVID-19 pandemic (Wosik et al., 2020). Lastly, Lee and DiGiuseppe (2018) report that interventions such as CBT may be more effective, however, this field requires further research with non-CBT interventions.

### The Process Model of Emotion Regulation

None of the studies included in previous reviews (Byrne and Coetzer, 2016; Iruthayarajah et al., 2018) used interventions based on a theoretically driven perspective, which relates to impaired ER as the likely mechanism of uncontrollable anger after ABI (Khan et al., 2003; Arciniegas and Wortzel, 2014; Salas et al., 2014; Caplan et al., 2015; Aboulafia-Brakha et al., 2016; Neumann et al., 2017; Winter et al., 2018). Although approaches such as CBT are widely used for anger management, they lack the focus on ER as the primary mechanism for moderating emotions (Salas et al., 2019). The Process Model of ER (Gross, 1998a,^2014^) is the only model that has informed ER studies after ABI (Salas et al., 2013, 2014; Rowlands et al., 2019, 2021), suggesting five classes of ER strategies: cognitive change, attentional deployment, situation selection, situation modification, and response modulation.

This article has two aims. Firstly, to discuss the efficacy of each strategy in individuals with ABI and/or non-clinical samples. Secondly, to recommend an anger management intervention for individuals with ABI, that includes at least two ER strategies. To demonstrate the difference between strategies, we discuss each in relation to a practical example of “arguing with a partner.”

#### Reappraisal

This strategy refers to altering the way an event is perceived (Gross, 2014). For example, after the argument, “we discussed practical ways of communicating better.” Reappraisal is one form of cognitive change, and is widely used to regulate discrete emotions in non-clinical samples (e.g., Nezlek and Kuppens, 2008) and individuals with ABI (e.g., Salas et al., 2013, 2014; Rowlands et al., 2019, 2021). A meta-analysis by Webb et al. (2012) investigated the efficacy of cognitive change, attentional deployment and response modulation in non-clinical samples. They reported a small-to-medium effect (*d*_+_ = 0.36) for reappraising emotional reactions. In comparison to other strategies such as response modulation and attentional deployment, evidence suggests that reappraisal is more effective when regulating negative emotions (McRae et al., 2010; Webb et al., 2012; Kalokerinos et al., 2015), and is preferred over attentional deployment for lower levels of affect (Gross, 2013; Van Bockstaele et al., 2020).

The few studies investigating reappraisal in ABI suggest that this strategy relies on executive elements that are often impaired in individuals with ABI (Salas et al., 2013, 2014; Dunning et al., 2016; Livny et al., 2017; Rowlands et al., 2019). For example, working memory, verbal fluency, and inhibition affected how long it took individuals with an ABI to produce a reappraisal, and working memory also affected the number of reappraisals produced (Salas et al., 2014; Rowlands et al., 2019).

Thus reappraisal is arguably more cognitively demanding than other ER strategies. However, findings suggest that once produced, they decrease the intensity of anger^3^ for individuals with ABI, in the same way as they do for neurologically normal individuals (Rowlands et al., 2019). While reappraisal appears challenging for individuals with cognitive impairment (Salas et al., 2014; Rowlands et al., 2019), it may still be a useful strategy, depending on the *level* of impairment. For example, individuals with milder cognitive sequelae may be suitable candidates, a line of enquiry that is worth exploring. Reappraisal therefore appears to be a suitable ER strategy for individuals with ABI who have cognitive impairment, because it is a widely investigated strategy, and one of the few strategies investigated in ABI, that has also demonstrated effectiveness for regulating negative emotions such as anger. In addition, it would be particularly relevant to compare reappraisal to another strategy that is less cognitively taxing.

#### Attentional Deployment

This strategy refers to moving attention away from emotion-evoking stimuli (Gross, 2014). For example, after the argument, “we chose to distract ourselves by watching a film.” Webb et al.’s (2012) meta-analysis report no effect size for attentional deployment as a strategy for emotional reactions in non-clinical samples. However, they suggest that the effectiveness of attentional deployment depends on strategy *type*. Two examples of attentional deployment are *distraction* (focusing on memories unrelated to the target emotion) and *concentration* (focusing on a task that elicits positive affect; Gross, 1998b). Findings from Webb et al.’s (2012) meta-analysis suggest that concentration is ineffective for emotional reactions (*d*_+_ = −0.26), whereas distraction is (*d*_+_ = 0.27).

Although findings suggest that decreased cognitive control impedes the execution of attentional deployment in neurologically normal individuals (Lohani and Isaacowitz, 2014; Wirth and Kunzmann, 2018), studies have yet to investigate the influence of executive functions on this strategy’s implementation and efficacy in ABI with cognitive impairment (Salas et al., 2019). Attentional deployment might be another suitable strategy. Firstly, compared to reappraisal, this strategy is *preferred* for regulating negative emotions in *older* adults (Scheibe et al., 2015). Secondly, distraction is preferred over reappraisal when regulating *high* levels of affect (Gross, 2013; Van Bockstaele et al., 2020). One explanation for this preference could be that these individuals find distraction less cognitively taxing. Overall, these findings, coupled with the fact that the majority of individuals who sustain an ABI *are* older adults (*M* = 47.8 years for TBI and *M* = 58.8 years for non-TBI; Colantonio et al., 2011), suggest that distraction is a strategy worth exploring.

#### Situation Selection

This strategy refers to choosing which situations to embrace or avoid, depending on the desired emotional outcome (Gross, 2014). For example, “we chose to go shopping at quieter times, as shopping during busy times leads to arguments.” Webb et al.’s (2012) meta-analysis does not include data for the effectiveness of situation selection, and this strategy has yet to be investigated in ABI. However, Webb et al. (2018) explored situation selection in two non-clinical samples, and propose two advantages: (1) it may be less cognitively demanding in comparison to other strategies; and (2) it does not require individuals to manage their emotions immediately. In terms of cognitive demand, Salas et al. (2019) suggest that situation selection may not be suitable for individuals with lesions to the ventromedial prefrontal cortex (vmPFC). It therefore seems inappropriate to include this strategy in a comparative anger regulation intervention for ABI, if individuals have sustained lesions to the vmPFC.

#### Situation Modification

This strategy refers to adapting one’s environment in accordance with a favorable emotional milieu (Gross, 2014). For example, “we agree on a grocery list before shopping, as shopping without one leads to arguments about what to buy.” Webb et al.’s (2012) meta-analysis does not include situation modification, and this strategy has yet to be investigated in ABI. Van Bockstaele et al. (2020) explored situation modification in a non-clinical sample, by allowing participants to choose between modification, distraction or reappraisal. Their findings suggest that situation modification is effective for regulating high levels of negative affect. While Livingstone and Isaacowitz (2015) propose that situation modification is not cognitively demanding, Salas et al. (2019) suggest that it might not be effective for individuals with lesions to the dorsolateral prefrontal cortex or vmPFC. It therefore seems inappropriate to include situation modification as part of a comparative anger regulation intervention for ABI.

#### Response Modulation

This strategy refers to changing an already elicited emotional response (Gross, 2014). For example, “we agree not to have the argument while we are shopping.” Webb et al.’s (2012) meta-analysis report a small effect for using response modulation for emotional reactions. Suppression, a type of response modulation, refers to purposely inhibiting an emotional reaction (Gross and Levenson, 1997). This strategy is particularly effective for inhibiting emotional *expression*, in comparison to inhibiting *thoughts* related to the emotion-inducing event.

Only one study has investigated response modulation in ABI. Salas et al. (2016) found that individuals with lesions to the right PFC and insula struggled to purposely inhibit or intensify the relevant facial expressions associated with positive emotions during a response modulation task. In terms of cognitive demand, they suggest that inhibitory control is associated with effectively suppressing positive emotions. Since response modulation relies on the ability to control the motor expressions associated with emotions, this strategy may not be effective for individuals with right frontal and insula lesions (Salas et al., 2019). Furthermore, evidence suggests that suppression is ineffective for regulating negative emotions (Kalokerinos et al., 2015). Taken together, it seems less optimal to include response modulation as part of a comparative anger regulation intervention for ABI.

## Discussion

While ER has been a popular research field (Gross, 2013), the study of ER after ABI is less well investigated (Salas et al., 2019). This article makes some recommendations about subjective experiences of anger, in relation to strategy number and type, shared decision-making, approaches to data analysis, and mode of delivery.

### Number of Strategies

The majority of studies investigate a single strategy (see Webb et al., 2012), while only a minority directly compare two or more (e.g., McRae et al., 2010; Kanske et al., 2011; Kalokerinos et al., 2015; Livingstone and Isaacowitz, 2015; Scheibe et al., 2015; Van Bockstaele et al., 2020). While single-strategy studies are noteworthy, good clinical practice would be to directly compare the efficacy of more than one approach, especially since some strategies rely on cognitive abilities often affected after ABI (Salas et al., 2014; Rowlands et al., 2019). In terms of comparative efficacy, the debate remains as to whether approaches stemming from a particular class of strategy are equally effective, or whether approaches from one class may be more effective than another (McRae et al., 2010; Kanske et al., 2011; Strauss et al., 2016).

### Strategy Type

Reappraisal is widely investigated, and the only strategy explored in ABI. Despite its cognitive demands, it has demonstrated evidence of regulating negative emotions by decreasing their intensity (e.g., Salas et al., 2013; Rowlands et al., 2019). Thus, it appears sensible to include reappraisal as *one* of the investigated strategies. A strategy that is less cognitively taxing, such as distraction, would present a good comparison, especially for regulating high levels of affect in older adults (Gross, 2013; Scheibe et al., 2015; Van Bockstaele et al., 2020). It is likely that the same lesion sites implicated in situation selection and modification are also implicated in reappraisal and distraction. However, evidence supports the efficacy and preference of the latter two strategies, and encourages prioritizing the investigation of these first. Furthermore, experimental conditions for strategies such as situation selection and modification might be challenging for individuals with ABI and cognitive impairment, if they are required to independently maintain their concentration during a computer-based task.

### Patient Agency and Choice

Shay and Lafata’s (2015) meta-analysis suggests that shared decision-making produced better affective-cognitive outcomes. Although the collaborative setting of treatment goals in neuro-rehabilitation has been considered in the literature (see e.g., McClain, 2005), active treatment *choice* by patients has, to our knowledge, not been empirically investigated in individuals with ABI. A novel intervention would give individuals with ABI the agency to c*hoose* a strategy that suits their strengths and circumstances.

### Data Analysis

The majority of studies in Webb et al.’s (2012) meta-analysis used quantitative measures, but a few used qualitative approaches. We suggest the use of a well-established quantitative measure of anger, with an additional qualitative component. An example of the former would be the State-Trait Anger Expression Inventory-2 (Spielberger, 1999), or the Overt Behavior Scale (Kelly et al., 2006), both of which include verbal *and* physical aggression subscales. An example of the latter would be semi-structured interviews on anger and the use of ER strategies. This combination is useful for two reasons: (1) it could yield insights into mechanisms behind the efficacy of interventions; and (2) it provides an alternative way to analyze data from underpowered clinical studies.

### Telemedicine

In light of the COVID-19 pandemic, delivery of many clinical services has shifted from in-person to virtually (Wosik et al., 2020), and telehealth has demonstrated advantages over in-person care^4^ (see Molini-Avejonas et al., 2015). Although there are limitations to virtual service delivery^5^ (Shaw et al., 2018; Cole et al., 2019; Mubaraki et al., 2021), evidence for the clinical effectiveness of telemedicine across a range of health sectors (see Bensink et al., 2006) supports virtually administered over in-person interventions (e.g., Rietdijk et al., 2020).

Furthermore, while evidence for telehealth in ABI is still emerging, results are encouraging, and show promise for future service delivery. For example, a patient with an ABI, who has been identified by their General Practitioner as someone with difficulties regulating anger, could be referred to an ER-based virtual intervention program. This program would consist of one-to-one Zoom meetings that focus on practical application of one or more ER strategies, with a homework component.

### Conclusion

The field of ER has grown dramatically over the last three decades, highlighting its importance for understanding emotions in both clinical and non-clinical populations. While there has been tremendous progress in certain areas of the field, ER as a rehabilitative tool after ABI remains under-developed. ER interventions have the potential to help individuals with ABI manage their lives, in areas where they and their loved ones have substantial difficulties. These interventions can also contribute to the understanding of the brain basis of managing anger, and the underpinning mechanisms of change.

## Author Contributions

JAW wrote the first draft of the manuscript. OHT and RC contributed to the writing, revising, and editing of the manuscript. All authors contributed to the design of the manuscript, and read and approved the submitted version.

## Conflict of Interest

The authors declare that the research was conducted in the absence of any commercial or financial relationships that could be construed as a potential conflict of interest.

## Publisher’s Note

All claims expressed in this article are solely those of the authors and do not necessarily represent those of their affiliated organizations, or those of the publisher, the editors and the reviewers. Any product that may be evaluated in this article, or claim that may be made by its manufacturer, is not guaranteed or endorsed by the publisher.

## Footnotes

1 The Global Burden of Disease Study (2017) identified stroke and road injuries as two of the top ten leading causes of early death worldwide (Institute for Health Metrics and Evaluation [IHME], 2018).

2 These difficulties include problems with relationships and social interactions, decreased social contact, loss of old friendships or difficulty creating new ones, and unemployment or issues with returning to work (Morton and Wehman, 1995; Shames et al., 2007; Benedictus et al., 2010; Grauwmeijer et al., 2012; Ma et al., 2014; Ponsford et al., 2014; Stocchetti and Zanier, 2016; Ruet et al., 2018; Salas et al., 2018).

3 As well as other negative emotions such as fear and sadness.

4 Such as access to services, greater outreach for individuals living in rural areas, and reduced to no travel and wait times (Molini-Avejonas et al., 2015).

5 Such as access to efficient internet, technical difficulties, diagnostic challenges due to the lack of physical contact, and negative perceptions about telecare (Shaw et al., 2018; Cole et al., 2019; Mubaraki et al., 2021).
